# One health analysis of antimicrobial resistance in *Escherichia coli* from humans, animals, and the environment

**DOI:** 10.1093/bioadv/vbag099

**Published:** 2026-04-03

**Authors:** Beatus Lyimo

**Affiliations:** School of Life Sciences and Bioengineering, Nelson Mandela African Institution of Science and Technology, Arusha, Tanzania

## Abstract

**Summary:**

Antimicrobial resistance (AMR) poses a critical public health threat, with evidence suggesting the transfer of resistance genes between humans, animals, and the environment. This study investigates the distribution of AMR genes, plasmid types, and the population structure of *Escherichia coli* isolates from humans, livestock, fish, and the environment in Tanzania, with a subset of data from Kenya, using a One Health approach. A total of 174 Whole-genome sequencing (WGS) data were analyzed to identify AMR genes and evaluate their population structure. The findings reveal widespread dissemination of AMR genes across all sources. Aminoglycoside resistance genes and β-lactam resistance genes were prevalent across all environments. Quinolone resistance mutations were detected in isolates from humans, livestock, fish, and the environment. Tetracycline resistance genes were found in humans, livestock, and fish. Plasmid types IncFIA, IncI1, and IncFII exhibited extensive cross-source sharing, with strong connectivity between human and livestock. Principal Component Analysis (PCA) revealed that *E. coli* isolates from Kenya formed a tight, distinct cluster, while other isolates were more dispersed. These findings emphasize the interconnected nature of AMR across human, animal, and environmental sectors and underscore the need for integrated surveillance under a One Health framework to monitor and control the spread of clinically significant AMR genes.

**Availability and Implementation:**

The genomic datasets analyzed in this study are available from public repositories. Bioinformatics analyses were conducted using established pipelines and standard tools for AMR gene detection, plasmid typing, and population structure analysis.

## 1 Introduction

Antimicrobial resistance (AMR) is a major global public health threat, contributing significantly to illness and death worldwide; however, its true magnitude remains not well understood (Naghavi *et al.* 2024, WHO 2025). In the year 2019, its estimated that over 4.71 million deaths have associated with AMR, including 1.14 million deaths attributed to bacteria AMR (Naghavi *et al.* 2024, WHO 2025). The impact of AMR-related morbidity and mortality is particularly severe in low- and middle-income countries (LMICs), where individuals are 1.5 times more likely to die from antimicrobial resistance (AMR) compared to those in high-income countries (UICC 2023). This disparity is even more pronounced among young children, most of whom would otherwise have gone on to live healthy lives. Among children under the age of five who die from AMR, 99.65% are in low- or middle-income countries (Baker *et al.* 2018, UNICEF 2023). The magnitude of the problem underscores the need for urgent and targeted actions to reduce the burden of AMR.

In 2019, the United Republic of Tanzania reported an estimated 12,500 deaths directly attributable to AMR and an additional 54,000 deaths associated with AMR-related infections [Institute for Health Metrics and Evaluation (IHME) 2023, Sartorius *et al.* 2024]. Moreover, Tanzania was ranked as the 32^th^ highest globally in terms of age-standardized mortality rates associated with AMR, among 204 countries analyzed in a global study [Institute for Health Metrics and Evaluation (IHME) 2023]. This ranking highlights the disproportionate impact of AMR in low- and middle-income countries, where healthcare infrastructure and access to effective antibiotics remain challenges. In 2016, Tanzania formulated the National Action Plan for AMR (2017–2022) in response to recommendations from the WHO and the Global Health Security Agenda Joint External Evaluation (WHO 2015, Neema *et al.* 2023). Following this, a comprehensive One Health AMR Surveillance Framework was developed to facilitate the establishment of AMR surveillance systems across human, animal, and environmental health sectors (United Republic of Tanzania 2018).

The 2022 Global Burden of Disease study highlighted *Escherichia coli*, *Staphylococcus aureus*, *Klebsiella pneumoniae*, *Streptococcus pneumoniae*, *Acinetobacter baumannii*, and *Pseudomonas aeruginosa* as the six primary contributors to AMR-related deaths, accounting for 73% of such fatalities in 2019 (Murray *et al.* 2022).

*E. coli* is commonly used as indicator for water contamination in most of the urban areas and while many strains are non-pathogenic, certain lineages of *E. coli* have evolved into major causes of infections, including gastroenteritis, urinary tract infections (UTIs), and sepsis. The burden of infections caused by pathogenic *E. coli* is exacerbated by the rise in AMR (Lyimo *et al.* 2016a; Subbiah *et al.* 2020), a lack of adequate sanitation, and limited access to healthcare.

Global collective action is required to strengthen AMR surveillance, promote the development of rapid diagnostic tools, and integrate advanced technologies such as whole-genome sequencing (WGS) to minimize the unnecessary use of antibiotics. The One Health approach is critical in this effort, as AMR spreads across human, animal, and environmental interfaces, necessitating coordinated interventions. WGS has transformed the study of bacterial pathogens by providing comprehensive insights into phylogenetic relationships, the presence of antimicrobial resistance genes (ARGs), and the detection of virulence (Manyahi *et al.* 2014, Hazen *et al.* 2018). The most common extended-spectrum beta-lactamase (ESBL) genes, including *bla_CTX-M_*, *bla_TEM_*, and *bla_SHV_*, confer resistance to third-generation cephalosporins and are frequently associated with mobile genetic elements such as plasmids, enabling their rapid dissemination across different hosts and environments (Nordmann *et al.* 2011). Integrating One Health principles into AMR surveillance enhances the ability to track resistance patterns, identify transmission pathways, and develop targeted strategies to mitigate its impact on public health, veterinary medicine, and environmental ecosystems (Peirano *et al.* 2010, Bailey *et al.* 2011, Accogli *et al.* 2013, Wu *et al.* 2014). One of the most significant contributions of WGS and bioinformatics tools is the ability to trace the movement of antibiotic resistance genes between different bacterial populations. This paper explores the use of the One Health approach to WGS data available in public repositories, focusing on *E. coli* strains circulating in East Africa to study examines the distribution of antibiotic resistance genes and genetic diversity of *E. coli* strains across human, animal, and environmental reservoirs.

## 2 Methods

### 2.1 Literature search strategy

A structured literature search was conducted to identify peer-reviewed studies reporting whole-genome sequencing (WGS) data on antibiotic-resistant bacteria from Tanzania, with a defined subset of comparative data from Kenya. The geographic focus on Tanzania (with Kenya included where relevant) was explicitly stated in the study objectives and abstract. The search was performed across major scientific databases and journals, including PubMed, Web of Science, and Google Scholar, using predefined keywords and Boolean combinations such as “antibiotic resistance in Tanzania,” “antibiotic resistance in humans in Tanzania,” “antibiotic resistance in livestock in Tanzania,” and “antibiotic resistance in the environment (soil and water) in Tanzania.” Equivalent search terms were applied to identify relevant studies from Kenya.

### 2.2 Criteria for study selection

Studies were included if they met all of the following criteria: (i) reported original research conducted in Tanzania or Kenya; (ii) focused on antibiotic resistance in bacterial isolates obtained from humans, livestock, or environmental sources (soil or water); (iii) employed WGS using recognized sequencing platforms; and (iv) provided accession numbers or sufficient metadata to allow retrieval of WGS data from public repositories. Studies were excluded if they were review articles, lacked genomic sequencing data, did not report antibiotic resistance outcomes, or were conducted outside the specified geographic scope.

For each eligible study, accession number(s) corresponding to bacterial genomes were extracted and used to retrieve WGS data from publicly accessible repositories, including the NCBI Sequence Read Archive (SRA) and GenBank. In addition, the *Escherichia coli* reference strain K-12 substr. MG1655 (Keseler *et al.* 2021), was included for comparative purposes. The final dataset comprises genomes aggregated from multiple independent BioProjects rather than a single cohort (Table 1). All retrieved datasets were subsequently subjected to downstream bioinformatic analyses as described below.

**Table 1:** Presents the list of accession numbers for the genomic sequences included in this study. These accession numbers correspond to the *E. coli* isolates analyzed across various sources, including human, livestock, and environmental samples.

SN	Accession	Source	Reference
1	PRJEB12361	Fish and environmental samples	(Moremi *et al.* 2016)
2	PRJEB12376	Humans in community settings	(Mshana *et al.* 2016)
3	PRJEB71714	Patients attending hospitals	(Kanje *et al.* 2024)
4	PRJEB32607	Faecal samples	(Muloi *et al.* 2022)

### 2.3 Data preprocessing

All data were checked for quality using fastqc v0.12.1 to generate individual report and MultiQC to put all reports together, then trimmed to remove low-quality sequences and adapters using Trimmomatic v0.39 (Bolger *et al.* 2014) before downstream analysis.

#### 2.3.1 Genome assembly

Paired-end reads were assembled into contigs using SPAdes genome assembler v3.15.5. Genome annotation was performed by the Prokka software v1.15.6 (Seemann 2014). Single nucleotide polymorphism (SNP) variant calling was performed using bcftools software v1.21 (Danecek *et al.* 2021). In brief the sequences were mapped to the reference genome *E. coli* str. K-12 substr. MG1655 to generate SAM files and then converted to BAM files and then sorted to produce sorted BAM file, then generate the mpileup. The bcftools was then used for variant calling to generate VCF files of each sample. The individual VCF file were merged to produce one merged vcf file using bcftools 1.21.

#### 2.3.2 AMR gene identification

SPAdes genome assembler v3.15.5 was used to generate contig sequence from each paired end sequence. The ResFinder Plus software v4.7.2 (Florensa *et al.* 2022) was used to identify AMR genes. In house pythone script followed by pheatmap function in R software version 4.3.1 were used to generate heatmat plots.

The ggVennDiagram function in R was used to show the distribution of shared and unique antimicrobial resistance genes, (ARGs) among five different sources.

#### 2.3.3 Population structure

To assess gene flow between *E. coli* isolated from human, livestock and environment (soil and water), genetic differentiation was estimated using the population structure was determined using principal component analysis (PCA) as implemented in PLINK1.9 (Purcell *et al.* 2007). To further examine the genetic relatedness of AMR strains, multilocus sequence typing (MLST v2.23.0) was performed in a Linux environment. The resulting allelic profiles were used to construct a network of related isolates, which was visualized in R using the ggraph and tidygraph packages.

## 3 Results

### 3.1 AMR gene identification and distributions

A total of 174 whole-genome sequences retrieved from the NCBI database were analyzed, revealing widespread distribution of AMR genes across environmental, fish, human, and livestock sources (Table 2). Resistance determinants for several antimicrobial classes including aminoglycosides, β-lactams, quinolones, and sulfonamides were detected across all sources, with β-lactam genes such as *blaTEM-1*, *blaOXA-1*, and *blaCTX-M-15* consistently present. Tetracycline resistance genes [*tet(A)* and *tet(B)*] were mainly observed in human, livestock, and fish isolates, suggesting potential links to antimicrobial use in animal production and environmental dissemination.

**Table 2:** Shows the distribution of detected resistance genes in selected antibiotics among *E. coli* isolates from humans, livestock, fish, and the environment.

Antimicrobial class	Environment	Human	Fish	Livestock (Kenya)	Livestock
**Aminoglycoside**	a*ac(3)-IId, aac(3)-IIe, aph(3'') -Ib, aph(6)-Id, aadA1, aadA5 (n = 6)*	*aac(6')-Ie/aph(2'')-Ia, aac(3)-IIe, aac(3)-IId, aac(3)-Ia, ant(2'')-Ia, aph(3')-Ia, aadA5, aadA2, aadA1, aph(3'')-Ib, aph(6)-Id (n = 11)*	*aac(3)-IId, aadA1, aph(3'')-Ib, aph(6)-Id, aadA2 (n = 5)*	*aph(3')-Ia, aadA1, aph(6)-Id, aph(3'')-Ib, aadA5, aadA2* *(n = 6)*	*aac(3)-IId, aac(3)-IIe, aph(6)-Id, aph(3'')-Ib, aadA5 (n = 5)*
**Beta-lactam**	*bla*_TEMp_C32T_, *bla*_TEM-1_, *bla*_SHV-11_, *bla*_SHV-1_, *bla*_TEM_, *bla*_OXA_, *bla*_OXA-301_, *bla*_CTX-M-15_, *bla*_OXA-1_, *bla*_CMY_, *bla*_CTX-M-55_ *(n = 11)*	*bla*_TEMp_C32T_, *bla*_TEMp_G162T_, *bla*_TEM-1_, *bla*_I_, *bla*_TEM-190_, *bla*_OXA_, *bla*_Z_, *bla*_R1_, *bla_NDM-5_*, *bla*_CTX-M-15_, *bla*_OXA-1_, *bla*_CTX-M-27_, *bla*_OXA-10_, *bla*_CMY-42_, *bla*_OXA-1181_, *bla*_DHA-1_, *mecA*, f*tsI_N337NYRIN, ftsI_I336IKYRI* *(n = 19)*	*bla*_TEM-1_, *bla*_CTX-M-15_ *(n = 2)*	*bla*_TEMp_C32T_, *bla*_TEM-1_, *bla*_OXA-1_ *(n = 3)*	*bla*_TEM-1_, *bla*_CTX-M-15_, *bla*_OXA-1_ *(n = 3)*
**Quinolone**	*qnrS1, qnrB1, qnrB48, gyrA_D87N, gyrA_S83L, parC_E84V, parC_S80I, parE_I529L, gyrA_S83A*, parE_I355T *(n = 10)*	*gyrA_D87N, gyrA_S83L, parC_S80I, parE_S458A, parC_E84G, parE_L416F, qepA4, parC_E84V, parE_I529L, parE_E460D, qnrS1, qnrS, qnrB4 (n = 13)*	*gyrA_S83L, parE_S458A, parC_S80I, gyrA_D87N, qepA4, parC_E84K, parE_L416F (n = 7)*	*qnrS1, parC_S80I, gyrA_S83L, gyrA_D87Y, gyrA_D87N, parE_S458T, qepA4 (n = 7)*	*qnrS1, gyrA_D87N, gyrA_S83L, parC_S80I, parE_S458A* *(n = 5)*
**Streptothricin**	*sat2 (n = 1)*	*sat2 (n = 1)*	*sat2 (n = 1)*	*sat2 (n = 1)*	– *(n = 0)*
**Sulfonamide**	sul2 *(n = 1)*	sul1 *(n = 1)*	sul2, sul1 *(n = 2)*	sul1, sul2 *(n = 2)*	sul1, sul2 *(n = 2)*
**Tetracycline**	*tet(A), tet(D)* *(n = 2)*	t*et(B), tet(A), tet(38), tet(K) (n = 4)*	*tet(B), tet(D) (n = 2)*	*tet(A), tet(B)* *(n = 2)*	*tet(A), tet(B)* *(n = 2)*

### 3.2 Heatmap and hierarchical clustering of AMR profiles

**Figure 1:**
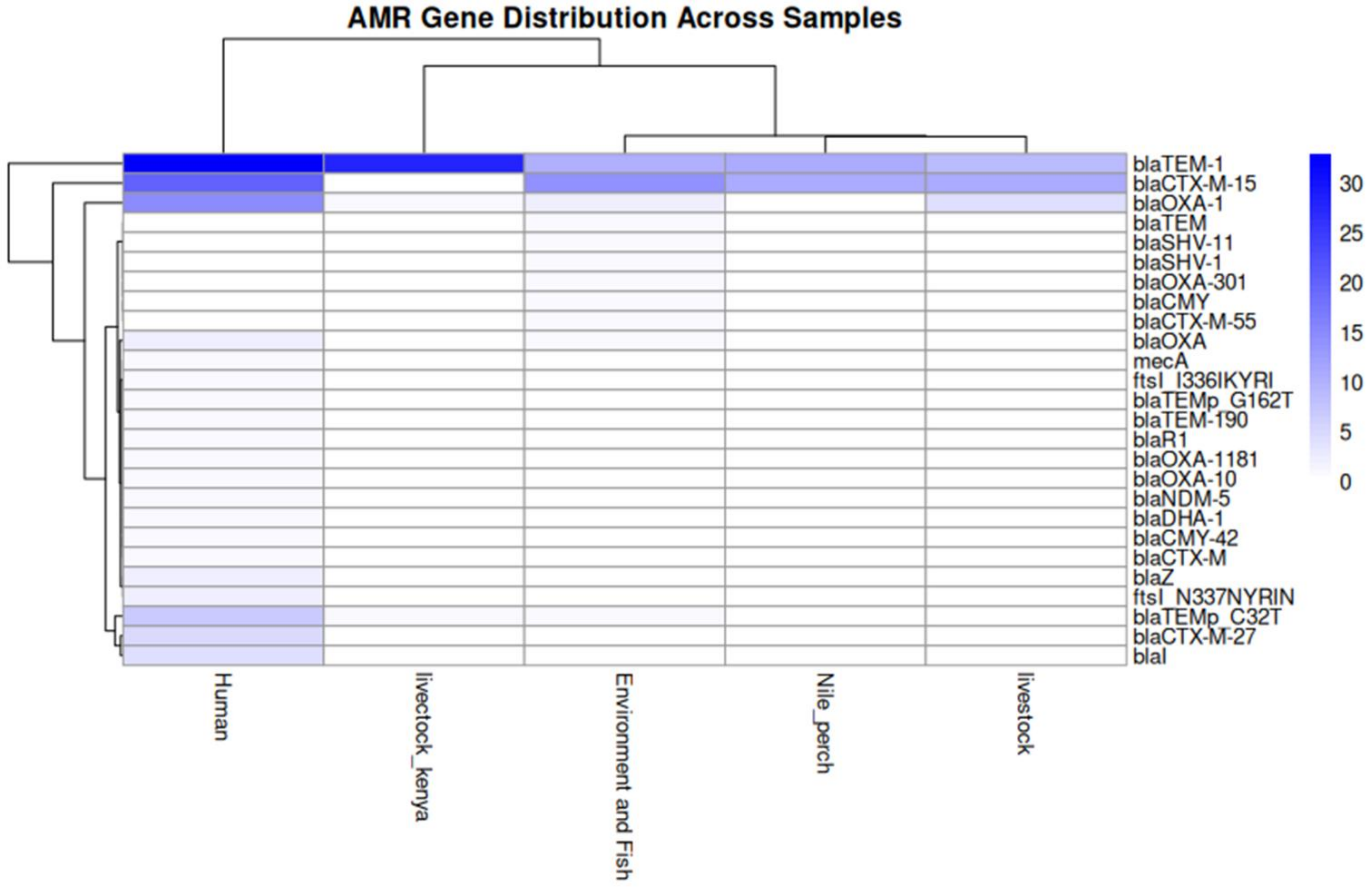
Heatmap plot of the relative abundance of ARGs in human, livestock, Nile pecrch, environment and fish from Tanzania and livestock from Kenya. Clustered each group ARGs in rows columns and source in columns.

Heatmap of the presence and absence of key AMR genes across the sample set (Fig. 1), showed the hierarchical clustering of isolates based on their resistance profiles reveals distinct groupings that align with their sources. Isolates from clinical settings form a minimal cluster from those obtained from environmental and animal sources. Additionally, isolates from livestock in Kenya cluster separately from other isolates, indicating potential geographic or host-specific variations in AMR gene distribution.

**Figure 2:**
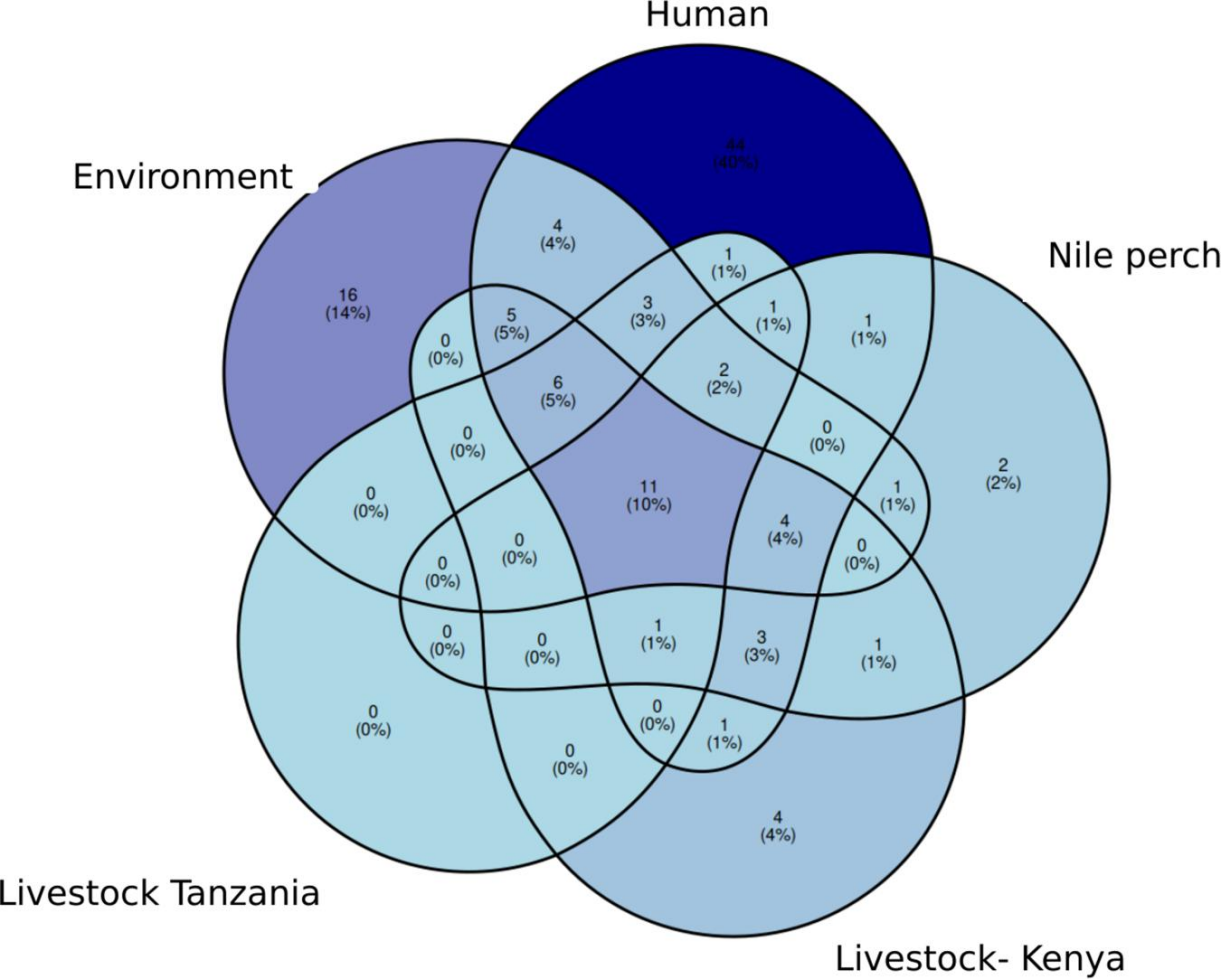
Venn Diagram of ARG distribution across different sources. Numbers in circles represent the count of unique and shared ARGs among sources, with percentages indicating relative proportions.

The Venn diagram illustrates the distribution of antimicrobial resistance genes (ARGs) across four ecological sources: humans, livestock (Tanzania and Kenya), fish, and the environment (Fig. 2). The numbers within each circle represent the count of unique and shared ARGs across these sources, with percentages indicating their relative proportions. Human isolates show the highest number of unique ARGs (44, 40%) while environmental samples contain 16 unique ARGs (14%). Shared ARGs among the four sources are limited, with only 11 ARGs (10%) found across all groups.

### 3.4 The plasmid network

**Figure 3:**
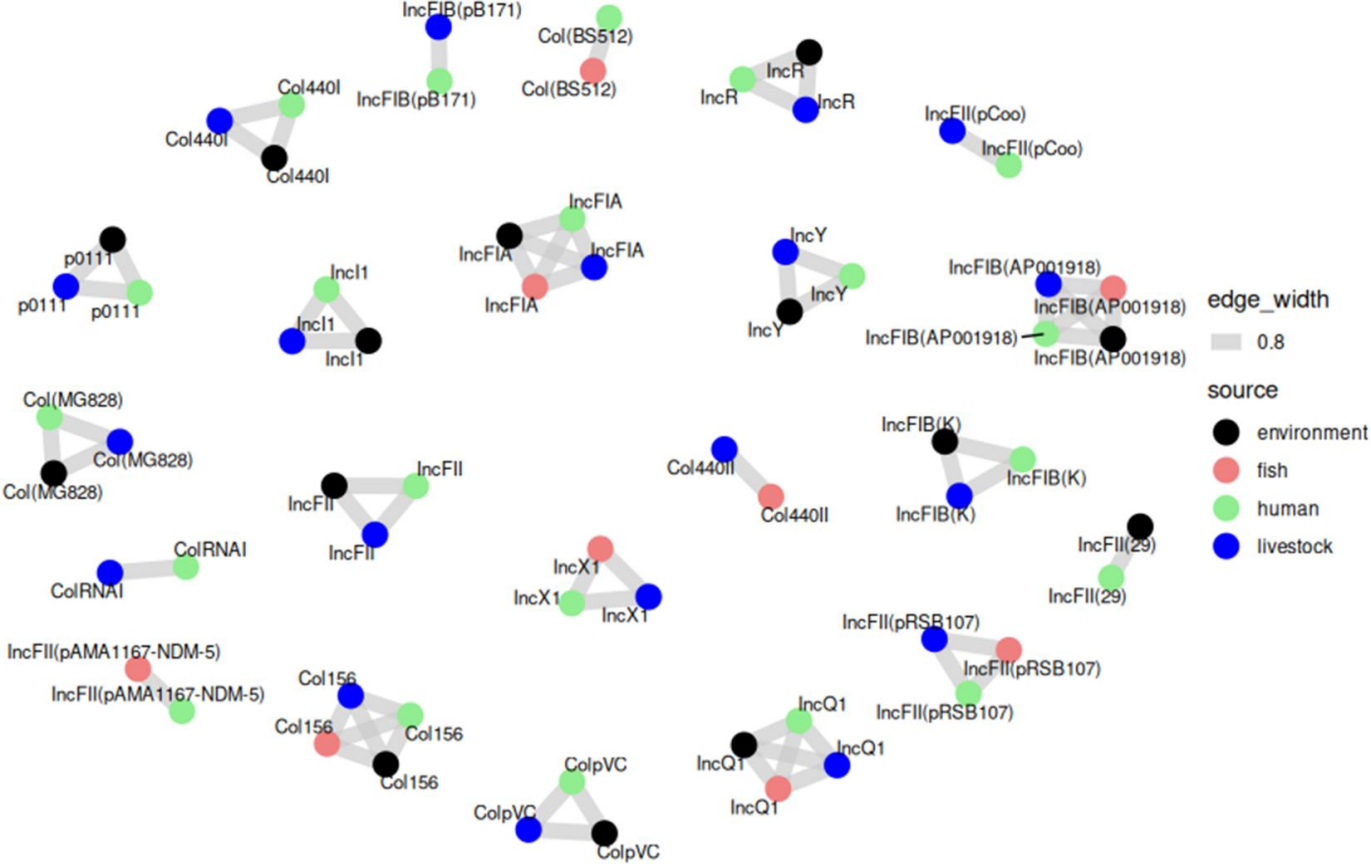
Plasmid Network of *E. coli* Isolates Across Human, Livestock, Fish, and Environmental Sources. Which showed the evidence of Cross-Source Horizontal Gene Transfer.

The plasmid network visualization depicts the relationships between plasmid replicon types detected in *E. coli* isolates from humans, livestock, fish (Nile perch), and the environment (Fig. 3). Node colors represent sources (human = green, livestock = blue, fish = red, environment = black), while edges indicate shared plasmid types, with thickness reflecting connection strength. Plasmid types IncFIA, IncI1, IncFII show the most extensive cross-source sharing. Human and livestock isolates exhibit the most significant connectivity. In addition, other replicon types, including IncFIB and Col, also demonstrated notable cross-source connectivity, although to a lesser extent than the dominant IncF groups. Environmental nodes show widespread but weaker connections.

### 3.5 Population structure

**Figure 4:**
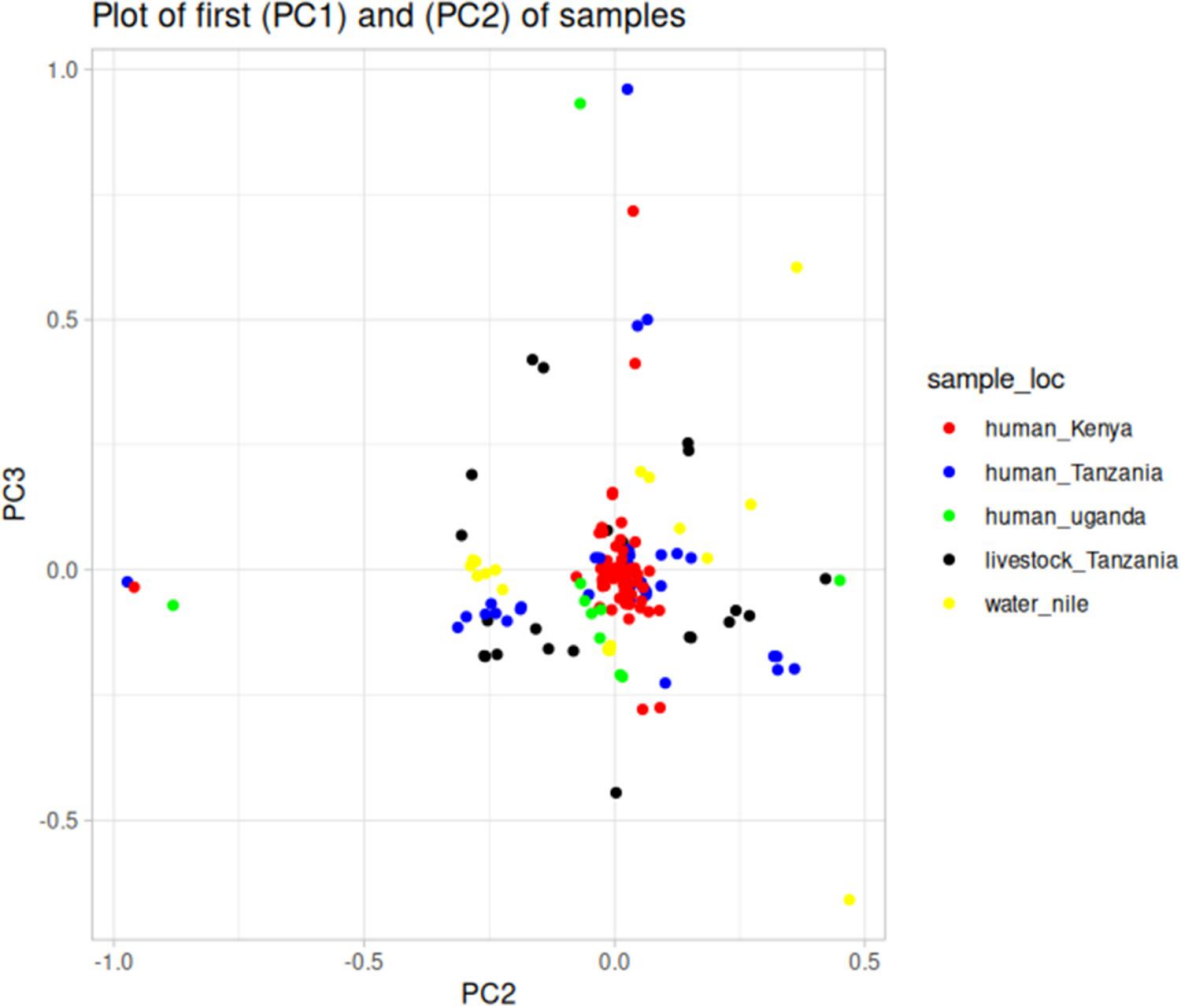
presents the principal component analysis (PCA) of *E. coli* isolates collected from various sources and locations.

The PCA analysis of *E. coli* isolates from human, livestock, fish and environment (Fig. 4) showed isolates from Kenya (grey) form a minimal distinct cluster, suggesting a shared genetic background or common selective pressures within this region. Other isolates from humans, livestock, and the environment are more dispersed across the PCA space.

### 3.6 Minimum spanning tree of cgMLST

**Figure 5:**
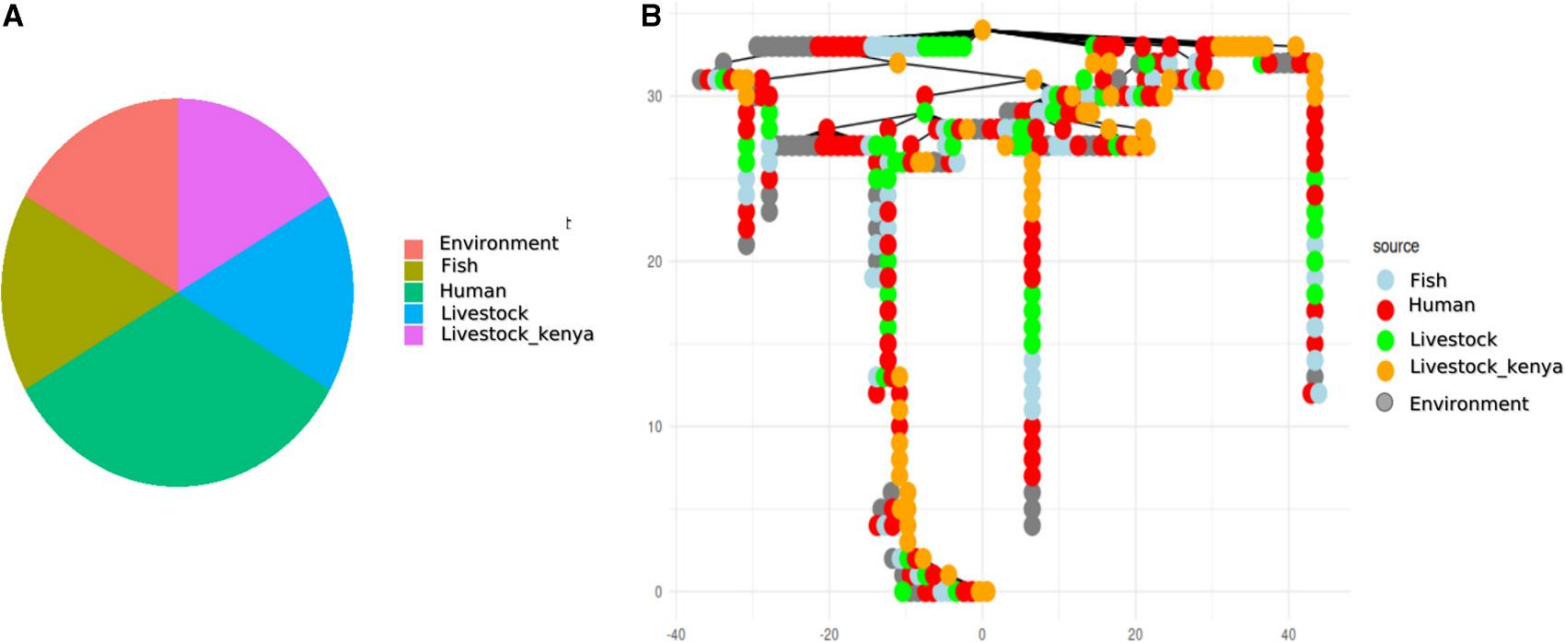
(A) The pie chart presents the proportional distribution based on allelic profiles from *E. coli* across different sources. (B) Minimum Spanning Tree (MST) based on core genome Multilocus Sequence Typing (cgMLST). Each node represents ST, and the edges indicate genetic relatedness. Nodes are colour-coded according to the source of isolation. The branching structure highlights potential evolutionary trajectories, with some clusters showing a dominant source while others appear mixed.

Results showed the pie chart which displays the proportion of ST from different sources. The sources include the environment, fish, humans, livestock, and livestock (Kenya). The distribution appears relatively even, with all sources contributing significantly. Livestock and human constitute a major proportion, highlighting their role in AMR transmission. Fish and environmental sources also contribute, reinforcing the multi-sectoral AMR dissemination (Fig 5A). Results showed also clustering patterns reveal that human isolates (red nodes) are closely related to livestock isolates (green, orange), suggesting potential transmission events. Environmental isolates (grey nodes) are distributed throughout the tree, implying their role as reservoirs or intermediates in pathogen transmission. Fish isolates (light blue) are interspersed, suggesting interactions between aquatic and terrestrial ecosystems. Multiple STs were shared between human and livestock isolates, including ST21, ST132, ST87, ST636, ST1120, ST681, ST532, ST666, and ST131, each represented by multiple isolates per source rather than single occurrences. Among these, ST21 and ST132 were the most frequently observed, occurring repeatedly across all host categories (Fig 5B).

## 4 Discussion

Bacterial resistance toward broad-spectrum antibiotics has become a major concern in recent years. The One Health approach has been widely advocated to enhance the understanding of the transmission dynamics of antibiotic-resistant bacteria from different sources (McEwen and Collignon 2018, Cella *et al.* 2023). Additionally, advancements in sequencing platforms and bioinformatics tools have significantly revolutionized the ability to trace the AMR transmission between humans, livestock, and the environment (Satam *et al.* 2023, Quitmeyer 2024). Furthermore, comparative analyses of different sequencing platforms and databases have been conducted to provide comprehensive analysis pipelines for defining AMR gene occurrence (Soni *et al.* 2021). These studies highlight the critical role of secondary data analysis in accurately identifying and characterizing AMR genes, thereby informing strategies to combat the spread of resistant pathogens. Collectively, these advancements demonstrate how secondary analysis of available sequence data could revolutionize our capacity to trace AMR transmission across various reservoirs. Therefore, this study leverages existing WGS data to elucidate the distribution and genetic relationships of antibiotic-resistant *E. coli* isolated from diverse sources within the One Health framework, encompassing humans, livestock, and environmental reservoirs, and the role of each source in the transmission of multidrug-resistant *E. coli.*

Results indicated a significant burden of antibiotic resistance genes across all sources. Among all antibiotic classes, aminoglycosides and β-lactams exhibited the highest prevalence across all samples. This finding is consistent with studies showing that resistance to β-lactams and aminoglycosides is increasingly common in human, animal, aquatic ecosystems and environmental reservoirs which, underlining the interconnectedness of human, animal, and environmental reservoirs in AMR transmission (Pormohammad *et al.* 2019, Gaşpar *et al.* 2021, Kiiti *et al.* 2021, Gemeda *et al.* 2023). Notably, the *bla_CTX-M-15_* gene was detected in nearly all samples, including isolates from fish and environment. This gene encodes a β-lactamase enzyme that confers resistance to third-generation cephalosporins, particularly ceftriaxone, cefotaxime, and ceftazidime. Reports from other studies have reported the occurence *bla_CTX-M-15_* in *E. coli* isolated from different sources ranging from human, livestock, and environment (Lyimo *et al.* 2016b, Moremi *et al.* 2016, Minja *et al.* 2021, Shawa *et al.* 2021, Mwakyabala *et al.* 2023). Based on these studies, *bla_CTX-M-15_* has emerged as the most prevalent ESBL gene across Africa; therefore, its detection in all environmental samples is not unexpected.

Resistance genes, such as *aph(3'')-Ib*, *aph(6)-Id*, *aadA1*, and *aadA2*, which are responsible for resistance to the aminoglycoside class of antibiotics, particularly streptomycin, have been shown to exhibit high prevalence across all sources (Shi *et al.* 2013, Zhang *et al.* 2023). The presence of these resistance genes in clinical isolates, as well as environmental and agricultural isolates, suggests a widespread dissemination of resistance mechanisms across various settings, including hospitals, communities, and agricultural environments.

In some isolate the study found occurrence of more than five different resistance gene occurring together. The environment, including soil and water, may act as an intermediary in the circulation of bacteria and resistance determinants, particularly through manure application and wastewater contamination. Human exposure could occur through contact with farm animals, handling or exposure to animal manure, contaminated water, aerosols, or the consumption of inadequately cooked animal products such as meat, eggs, and milk. These pathways represent plausible routes of exposure rather than direct evidence of transmission (Jaja *et al.* 2020, Zhang *et al.* 2023). Moreover, the high prevalence of these key resistance determinants, such as bla_**CTXM15**_ and aminoglycoside-modifying genes, may reflect regional antimicrobial selection pressures in livestock and healthcare settings rather than global dissemination trends. This information is very critical as applying one health in the control of AMR transmission.

The venn diagram results showed human exhibits the highest number of unique antimicrobial resistance genes (44, 40%), suggesting that medical antibiotic use plays a dominant role in resistance selection. These resistance determinants could be driven by excessive antibiotic prescriptions, self-medication, or hospital-acquired infections. The resistance determinant genes can find there ways back to animals or environment.

The environment category contains a substantial number of unique ARGs (16, 14%), emphasizing the importance of wastewater contamination, aquaculture antibiotic use, and environmental persistence of resistance genes. Since many resistance genes in the environment are derived from human and livestock sources, pollution control and water treatment strategies are critical for AMR mitigation. The overlapping ARGs between this category and others suggest the potential for ARG spillover into aquatic ecosystems, contributing to horizontal gene transfer (HGT) between bacterial communities (Guo *et al.* 2017, Napit *et al.* 2025).

Livestock samples from Tanzania and Kenya exhibit fewer unique ARGs and relatively low overlap with Nile perch. This suggests that direct transmission between these reservoirs is limited. However, ARG exchange may still occur through shared environmental exposure, such as contaminated water sources, runoff from farms, or use of animal waste in aquaculture. The presence of some common ARGs (e.g. 11 shared ARGs, 10%) indicates potential indirect transmission pathways. Differences between livestock in Tanzania and Kenya indicate potential geographical, management, and antibiotic use policy variations. These findings reinforce the need for harmonized antimicrobial use policies in East Africa, focusing on standardized surveillance and interventions across borders. The where further confirmed by heatmap results which showed illustrated the distribution of antimicrobial AMR genes across various sample sources, including livestock from Kenya, human, livestock, fish and environmental (water and soil) and fish samples from Tanzania. The hierarchical clustering provides insights into the similarities between AMR gene profiles across sample categories and the co-occurrence of resistance genes. This result aligns with other studies which showed co-occurrence of antibiotic resistance gene across different sources (Altayb *et al.* 2022, Inda-Díaz *et al.* 2023).

The plasmid network visualization reveals important insights into *E. coli* populations from different sources, including humans, livestock, fish, and the environment. This finding shows that *E. coli* from all sources harbor IncF plasmids, with IncFIA, IncI1, and IncFII notably widespread across multiple sources, indicating their pivotal role in facilitating gene exchange. These plasmids are known to carry genes related to antibiotic resistance, virulence factors, and other traits that enhance bacterial survival and adaptation in diverse ecological niches (Lyimo *et al.* 2016c, Rozwandowicz *et al.* 2018a, Pankok *et al.* 2022, Chen *et al.* 2024).

The PCA results offers valuable insights into the complex interactions between humans, livestock, and the environment, particularly with regard to *E. coli* transmission and AMR dynamics (McEwen and Collignon 2018, Velazquez-Meza *et al.* 2022). The broader dispersion of the isolates from humans, livestock, and the environment across the PCA plot indicates significant variability within these groups, pointing to a heterogeneous nature of *E. coli* populations in different ecological niches. This could reflect a variety of factors, including the diversity of *E. coli* strains in different host species, environmental influences such as water quality or soil conditions, and the role of human behavior (e.g. antibiotic use, hygiene practices) in shaping microbial populations.

The analysis of *E. coli* STs across humans, livestock, fish, and environmental sources revealed a consistent distribution of STs in our dataset, with ST21, ST132, ST2, ST666, ST681, and ST1120 among the most prevalent across all sources studied. The observation of identical or overlapping STs across ecological compartments suggests widespread dissemination of specific *E. coli* lineages in multiple niches, consistent with reports from other One Health genomic investigations that have documented shared STs among humans, animals, and environmental samples, indicating interhost and environmental connectivity of clonal groups and resistance determinants (Musicha *et al.* 2026). Such overlaps are hallmarks of ongoing ecological transmission and highlight the potential for horizontal gene flow.

However, interpretation of ST sharing and inferred transmission dynamics should be made with caution, as differences in sample size across sources (Fig. 5A) may bias the apparent frequency and diversity of STs. Nevertheless, the consistent detection of identical STs across humans, livestock, fish, and environmental samples provides strong evidence for interconnected *E. coli* populations and cross-reservoir circulation at the human–livestock–environment interface, reinforcing a One Health transmission framework.

Overall, the results highlight the need for integrated One Health surveillance strategies to monitor and mitigate the spread of AMR across human, animal, and environmental reservoirs. The presence of clinically relevant resistance genes in non-human sources raises concerns about zoonotic transmission and the role of environmental compartments as AMR reservoirs.

## 5 Conclusion and recommendations

This study demonstrates a high prevalence and wide distribution of ARGs in *E. coli* isolated from humans, livestock, fish, and the environment, reinforcing the interconnected nature of AMR within the One Health framework. Key resistance genes were consistently detected across all sources, while plasmid types IncFIA, IncI1, and IncFII were identified across multiple sources. The observed connectivity between human and livestock isolates suggests possible zoonotic or bidirectional transmission pathways. PCA further revealed substantial genetic diversity among isolates, likely driven by ecological, environmental, and host-specific selective pressures.

Addressing these challenges requires a multidisciplinary approach integrating enhanced surveillance, improved wastewater management, antimicrobial stewardship, and advanced genomic and bioinformatics techniques. Implementing these strategies within the One Health framework is essential to mitigate the spread of antimicrobial resistance across ecosystems.

## 6 Limitations

This study has several limitations. First, the sampling coverage and number of isolates were limited to specific regions, which may not fully capture the national distribution of antimicrobial resistance. The analysis focused solely on *E. coli*, and inclusion of additional bacterial species could have provided a broader understanding of resistance dissemination. Furthermore, the study relied on genomic data without complementary phenotypic susceptibility testing or functional validation of detected genes, which may limit interpretation of their actual resistance expression and transfer potential. Lastly, incomplete metadata such as antibiotic usage history and environmental parameters constrained the capacity to link resistance patterns to specific drivers.

## 7 Future directions

Future studies should aim to expand geographic and sample coverage to capture the national distribution of antimicrobial resistance and include additional bacterial species to provide a broader understanding of resistance dissemination. Integrating phenotypic antimicrobial susceptibility testing with genomic analyses will help confirm the expression and transfer potential of detected resistance genes. Collecting detailed metadata, including antibiotic usage, animal husbandry practices, and environmental parameters, will improve the ability to link resistance patterns to specific drivers. Longitudinal monitoring and investigation of resistance mechanisms, such as plasmid-mediated gene transfer, will further elucidate the dynamics of antimicrobial resistance across humans, animals, and the environment.

## Data Availability

All analysis codes used in this study have been deposited in a publicly accessible GitHub repository and is available at: https://github.com/beatusmodest/AMR-in-One-health
